# On the Thymus Gland

**Published:** 1835-04-01

**Authors:** W. F. Bow


### *On the Thymus Gland.* By W. F. Bow, m.d.

*To the Editor of the Medical Quarterly Review.*

Sir: In your "Retrospect of the late Improvements in Medicine and Surgery, &c.," to be found in the sixth number of the Medical Quarterly, mention is made of the conjecture of Sir Astley Cooper as to the use of the thymus gland, and you say that the same conjecture was previously published by me. So far as the thymic liquor is concerned, our conjectures agree, but not our notions as to the design of the gland. Sir Astley thinks it not improbable that the gland is designed to prepare the thymic liquor; whereas, I do not think, that the main design of the gland is to prepare this liquor, but the thymus I regard as an organ formed for the purpose of reserving, during the fœtal state, the nervous influence which is destined to flow to the respiratory apparatus after birth: this is the main object, the main design of its formation.

As the copies of my publication were very limited in number, permit me, through your pages, to promulgate further my opinions as to the uses of this almost forgotten viscus. Attention to it, since the publication of Sir Astley's works, seems in some measure rekindled; a little agitation, therefore, may elicit facts useful either in destroying or supporting conjecture.

According to my ideas of nervous action and of the distribution of nervous influence, there must necessarily exist in the fœtus an organ having the power of accumulating the nervous energy required by the lungs at the moment of birth, and which can, without detriment to the animal, by the sudden cessation of its function, yield up this energy. This necessity I deduce from the fact that we cannot increase the nervous activity of any one part of the body, without diminishing it in corresponding proportion in some other part. At the moment of birth, an immense nervous force is suddenly required by the chemical and mechanical respiratory apparatuses, and it is as suddenly furnished: whence then is it derived? It cannot be derived from any organ or structure whose function is necessary to the health or life of the respiring animal. Necessity, then, requires some contrivance in fœtal life which shall accumulate a power, the real use of which is prospective, and which, if not so consumed, would destroy the organization of the lungs long before the period at which the exercise of their functions becomes necessary. Do we know of any contrivance, of any organ, always existing in the perfect fœtus, that can yield its vitality at the birth of the child, without danger to the child, owing to the cessation of its function? We know that the function of the thymus ceases at birth, because soon after birth its lobes become thinned by absorption, and its reservoirs begin to be obliterated. Sir Astley Cooper says he injected the gland in a child of one month, and found that the lobes had become quite thinned by absorption, although the reservoirs in part remained; but even one of these was partially obliterated. In a child of four months, the reservoir was very small, and broken into several portions; and the weight of the glands, which should have been in the fœtus of nine months 240 grains, was only forty-five grains, or about five times less than in the fœtal state.

At birth we find a set of organs called on, for the first time, to exercise their functions, the agency requisite being nervous power: at the same moment we find an organ ceasing to perform any function, becoming paralyzed and dying, evidently owing to its being deprived of nervous power. The power lost is of the same nature as the power acquired; we must therefore presume that either the nervous power of the one organ is annihilated, and that of the others created, at the same moment, or that there is a transfer. Is not the latter supposition the more probable?

Such is the main object for which this gland exists. But as so much nervous influence must be determined to the gland, I thought that it must also be endowed with the power of secretion. I therefore referred to what was known of the anatomy of the gland before Sir Astley's satisfactory account was published, and learned that vessels resembling lymphatics passed from it to the thoracic duct, and that the gland and these vessels often contained a milky fluid resembling chyle. This fluid must be useful to the fœtus; and, as it is poured into the thoracic duct, thence to be circulated with the blood, its use, I concluded, could only be that of enriching the circulation, and furthering nutrition.

I know not if comparative anatomy can furnish direct proof that the thymus be mainly designed to act as a diverticulum to the nervous influence which, after birth, is intended to flow to the lungs; but it furnishes facts which prove that similar contrivances do exist in the fœtus for the purpose of expending nervous influence, the use of which is also prospective. The supra-renal capsules in the human fœtus are evidently mainly contrived to act as a diverticulum to the nervous influence intended to effect the secretion of urine after birth; this is directly proved by reference to comparative anatomy.

In the human fœtus the capsules are as large as the kidneys themselves; after birth, they dwindle in comparative size, until, in the adult, the kidneys exceed them by fifteen times. Thus, like the thymus, they are greatly developed, at birth; like it, they are largely supplied with nerves; like it, they dwindle after birth; and, like it, too, they are absent in children born without a brain.

The kid, when born, has a capsule one sixteenth the size of the kidney, which is nearly its relative size in the human adult. The capsule of the lama of a day old, Cuvier found to be only one hundredth of the size of the kidney. Comparing those animals, then, with the human subject, it may truly be said that they do not possess *fœtal* capsules, and that therefore, during the fœtal state, the nervous influence which effects the secretion of urine is not diverted from the kidneys. With these animals it is not necessary that the nervous power should be so diverted; for they are provided with an allantoid, so that the secretion of urine goes on before birth, without danger either to the mother or fœtus: fœtal capsules to them would be as superfluous as would an allantoid to the human subject.

Since we find an allantoid where the capsules are small, and none where they are large, we can come to no other conclusion than that the great development of them in the human subject renders the presence of such a membrane unnecessary; and the only way in which they can render it unnecessary, is by diverting from the kidneys that power which otherwise would produce the fluid that the allantoid is furnished to receive. The existence of an allantoid where the capsules are small, (or, as I said before, where fœtal capsules are wanting,) proves, beyond a doubt, that their principal use in the human fœtus is that which I have assigned to them; and, if a particular contrivance be necessary to consume the comparatively small force of nervous influence afterwards required for the secretion of urine, how much greater must be the necessity for a similar design to consume the immense force of the same influence destined to flow to the respiratory apparatus at birth.

The stomach, too, has its diverticulum—the spleen; but, as the process of digestion is not in constant operation after birth, the spleen does not degenerate: a diverticulum is still required when the stomach is inactive. When digestion in the stomach is over, the nervous influence, which effected the secretion of the gastric juice, reverts to the spleen: hence it increases in size, in obedience to a law of nervous action, of which I have endeavoured elsewhere to show the existence. When the chemical nerves of a secreting organ become active, or assume a positive state, the nerves conveying the contractile power to its arterial structure assume an opposite or negative state: hence distention of that structure is permitted, and the volume of the organ is consequently increased. Now, if we can fix upon a class of animals where the lungs after birth are, like the stomach, not in constant operation, we are prepared to find, in such animals, the thymus in a condition capable of again becoming a diverticulum during the inactivity of the lungs; and, as in the case of the spleen during the inactivity of the stomach, an increase in its size.

In hybernating animals, the lungs are not in constant operation: during hybernation, respiration is suspended. According to Dr. Hall, we cannot then detect any movements of breathing; the air of the pneumatometer, in which the animal is confined, is scarcely changed; the temperature of the animal is the same as that of the atmosphere; it is capable of supporting, for a great length of time, the entire privation of air, and the blood throughout the body is of the venous character. During this suspension of respiration, what is the condition of the thymus gland? It acquires an increase of size, exactly after the fashion of the spleen, during the inactivity of the stomach. In your Number for October 1834, you mention the increase of size of the thymus during hybernation, and you say that my supposition as to the main design of this organ is curiously confirmed by this fact. When I published I was not aware of this increase; but, when I afterwards saw it alluded to in an analytical notice of M. Hangsted's work, in the Medico-Chirurgical Review, I could not avoid hailing it as, at least, strong evidence in favour of my opinion of the gland's physiology.

From the same article I learn that Dr. Kopp is the author of a memoir on one form of asthma occurring in infants, which he traced to a hypertrophy of the thymus gland; the result of this morbid state being a certain degree of compression on the trachea and bronchi. The writer of the article in the Review says,

"The dyspnœa, we are told, usually occurs in paroxysms, and these are more frequent when the infant is sucking, or has just awakened from sleep, than at other times. The explanation of this is easy: during sleep the inspirations are less profound, and, the lungs being therefore less expanded, more room is left for the enlarged thymus. Whenever the child awakes, it suddenly draws in a deep breath; the thymus resists the full dilatation of the lungs: and a paroxysm of dyspnœa is thus induced. A fit of crying has nearly the same effect; and, in short, any effort which is accompanied by a sudden and rapid inspiration."

Before commenting on the above passage, I shall transcribe some more from the pages of the Review: by doing so here, I shall be able to give my remarks more connectedly.

"The mere circumstance of the thymus being found enlarged, in those cases alluded to by Dr. K., is not sufficient, in itself, to justify the conclusion that all the asthmatic symptoms are induced by the compression thereby exerted on the respiratory organs.

"It is not common to find an hypertrophied state of the gland alone, and unassociated with some lesions of other organs, especially of the heart and lungs. Such being the case, we can readily understand the cause of the respiratory distress; and it may reasonably be disputed, whether the enlargement of the gland is the cause or the result of the other lesions. Caspari and Pagenstecher have been led, by their investigations, to regard the increased volume of the thymus, in those cases included under Kopp's "asthma thymicum," as induced by, or as the consequence of, the dyspnœa; the true cause of which is, in their opinion, an affection of the par vagum. We cannot, however, reasonably doubt that the thymus, when it has acquired a considerable enlargement, must, of a necessity, offer some impediment to the free dilatation of the lungs, just in the same way as the liver, spleen, &c., when their dimensions are preternaturally increased, give rise to symptoms of distressed breathing. One difficulty, not easily explained, will readily suggest itself to the reader. How is it that the dyspnœa comes on in paroxysms, when the exciting cause is not an occasional, but a permanent one? Surely there is no reason for believing that the symptoms are referable to a periodic congestion of humours in the gland. The following case is important, as it shows that an abnormal enlargement of the thymus is not always accompanied with dyspnœa.

"A girl, seven years of age, was admitted into the hospital at Copenhagen, in October, 1830. For the preceding six months she had been afflicted with severe headaches, to which were now added sickness and occasional vomitings. The looks of the child were heavy and dull, and indicated, in connexion with other symptoms, the existence of hydrocephalic disease. The breathing had been all along, and still continued to be, quite easy and unobstructed. * * The patient died on the sixth day after her admission. On dissection, there was found a considerable effusion into the cerebral ventricles. The thymus gland was of unusual dimensions, and was much larger than in any one case of asthma thymicum related by Dr. Kopp himself: it weighed five ounces. The right lobe, which was the one most enlarged, extended in front of the right lung, to which it adhered as far as the angle of the ribs: it measured four inches in length, two in breadth, and was nearly an inch and a half in thickness. Its texture had become degenerated into a granular or tuberculous mass, which presented no traces of any cells or of lobules. The upper lobes of the lungs also exhibited tuberculous changes. The thyroid gland and the capsular supra-renales were normal.

"Meckel (primus) describes the history of a soldier, twenty-six years of age, who had long suffered from paroxysms of dreadful anxiety and dyspnœa: at first their recurrence was at considerable intervals, but afterwards they became much more frequent. In the upper part of the cavity of the anterior mediastinum, there existed two large lobes of the thymus; each of these was three and a half inches long, and from six to ten lines in breadth; their texture was nearly the same as in the fœtus; they were cellular, and might be distended by blowing air into them; several considerable-sized arteries and veins, derived from the internal mammaries and inferior thyroids, were distributed upon their substance; their colour was a reddish white; very little adipose matter existed in the surrounding cellular substance."

It would appear, then, that there are exceptions to the general law; that in some cases the thymus does not diminish, but, on the contrary, it increases in size; and that the individuals in whom this irregularity exists are subject to obstructed respiration, occurring in paroxysms. Admitting that a preternatural enlargement of the thymus may offer some impediment to the free dilatation of the lungs, the dyspnœa recurring in paroxysms admits not of this mechanical cause. The cause is that, whilst the thymus retains its original organization, there remains a tendency to resume its secreting function, or, at least, to recover the nervous influence it yielded to the lungs, when respiration commenced. And at what times do these paroxysms most frequently occur? "When the infant is sucking, or has just awakened from sleep:" at the very times when, if I may so express myself, the lungs are most off their guard; when the inspirations are less profound. If a paroxysm occur when the child has just awakened, I suspect that the commencing paroxysm is the cause of the sleep being broken, when a child is awoke by a sudden attack of croup. When a child sucks, the inspirations are frequent, and therefore cannot be profound; if he be a hearty sucker, it may be said that breathing to him is of secondary consideration,—he has no time for it.

I have transcribed the Copenhagen case and Meckel's case in full, because I think much information is to be gained from a comparative view of them. In the first we find the gland enormously enlarged: the measurement of the right lobe only is given, yet it would occupy more space than the lobes, the measurement of which is given by Meckel. In the Copenhagen case, the "breathing had been all along, and still continued to be, quite easy and unobstructed." In Meckel's case, the patient "had long suffered from paroxysms of dreadful anxiety and dyspnœa." Since, in the case of the greater enlargement of the gland, the breathing continued easy and unobstructed, we cannot blame the size of the gland for the dyspnœa in the other case, more especially as that dyspnœa was not constant. In the Copenhagen case, the texture of the gland "had become degenerated into a granular or tuberculous mass, which presented no traces of any cells or of lobules;" a disorganized mass, which could not interfere with the functions of the lungs, by resuming or by having a tendency to resume its former function. In Meckel's case, the texture of the lobes was "nearly the same as in the fœtus: they were cellular, and might be distended by blowing air into them; several considerable-sized arteries and veins, derived from the internal mammaries and inferior thyroids, were distributed upon their substance; their colour was a reddish white." Morbid anatomy could not furnish a better proof of the soundness of my views as to the design of the thymus, than this case. The gland increases in size as the body increases; it retains its original structure, and a tendency to resume its original function. In the fœtus it was a diverticulum; at birth, it yielded the nervous influence required by the lungs, but, perhaps retaining a part, it did not degenerate, and ever after showed a tendency to regain the whole; the attempts to do so causing dyspnœal paroxysms.

I begin to suspect that, in children who die of croup, the thymus will be found larger than it ought to be, owing to its not yielding to the lungs at birth all its nervous influence, and therefore, having a tendency at times, when accidental causes create irregularity in the distribution of nervous influence, to resume its original function; and that this tendency continues until the age at which the thymus becomes nearly obliterated: if this be the case, we have the reason why croup is seldom observed after the age of twelve.

"According to the researches of some pathologists, an abnormal development of the thymus has, in numerous instances, induced sudden and fatal suffocation in very young infants." If the thymus be a diverticulum in the fœtus, and if it can again divert nervous influence from the lungs after birth, we need look for no other explanation of these facts.

W. F. Bow.

*Alnwick; February* 12, 1835.

